# The Association between *Helicobacter pylori* Infection and Irritable Bowel Syndrome: A Meta-Analysis

**DOI:** 10.3390/ijerph17072524

**Published:** 2020-04-07

**Authors:** Yun-A Kim, Yoon Jeong Cho, Sang Gyu Kwak

**Affiliations:** 1 Department of Family Medicine, Daegu Catholic University School of Medicine, Daegu 42472, Korea; yuna815@dcmc.co.kr; 2 Department of Medical Statistics, Daegu Catholic University School of Medicine, Daegu 42472, Korea; sgkwak@cu.ac.kr

**Keywords:** *H. pylori*, irritable bowel syndrome, functional dyspepsia

## Abstract

The association of *Helicobacter pylori* (*H. pylori*) infection with functional dyspepsia has been well studied. However, the data on the relationship between *H. pylori* infection and irritable bowel syndrome (IBS) are conflicting. This study aims to elucidate the association between *H. pylori* infection and IBS. PubMed, Cochrane Library, CINAHL and SCOPUS databases were searched to identify eligible English articles published up to December 2019. Cross-sectional studies, case–control studies and cohort studies reporting both prevalence of *H. pylori* infection and IBS were selected for the detailed review. The pooled odds ratio (ORs) and their 95% confidence interval (CI) were calculated. A total of 7269 individuals in four cross-sectional studies and six case-control studies were included. The prevalence of *H. pylori* infection ranged from 12.8% to 73.4% in the control group, and 9.7% to 72.1% in the IBS group. The combined OR for *H. pylori* infection was 1.10 (95% CI: 0.93–1.29, I^2^: 37.5%). In a subgroup analysis of IBS defined according to Rome criteria, the OR for *H. pylori* infection was 1.10 (95% CI: 0.93–1.30, I^2^ = 31.7%). In this meta-analysis, *H. pylori* infection was not significantly associated with IBS. Well-designed studies are needed to identify the relationship between *H. pylori* infection and IBS.

## 1. Introduction

Irritable bowel syndrome (IBS) is a chronic functional disorder which is characterized as recurrent abdominal discomfort or pain accompanied by changes in bowel habit or defecation [1,2]. Although its prevalence has varied according to diverse diagnostic criteria, 10%–20% of western populations have experienced IBS-like symptoms [3,4,5], which can lead to decreased qualify of life [6,7], and many of them seek alternative treatment due to dissatisfaction with the traditional medical therapy [5].

*Helicobacter pylori (H. pylori)* is a Gram negative bacterium which is found on the surface of the gastric epithelium [8]. *H. pylori* is so common that about 50% of world population has been infected with *H. pylori*. Among these infections, 1%–15% develop into certain gastrointestinal diseases, such as peptic ulcers, gastric cancer and gastric mucosa-associated lymphoid-tissue (MALT) lymphoma [8,9]. During the last few decades, *H. pylori* infection has been proposed to be associated with a range of extra-digestive manifestations, ranging from vascular diseases to hepatopathies and colorectal carcinoma [10,11]. In addition to these *H. pylori*-related diseases, the association with functional dyspepsia has been also reported [12,13,14].

Similar to functional dyspepsia [15], alterations in gut epithelium and microbiome which may originate from psychological disturbance [5], chronic mucosal inflammation [16] or visceral hypersensitivity [17] have been suggested as possible pathophysiologies of IBS. In regard to gastrointestinal infection and inflammation, the association between *H. pylori* and IBS has been studied, though the results are inconsistent [16,18,19,20,21,22,23]. In a large observational study in China, neither dyspepsia nor IBS was associated with *H. pylori* infection [21], and *H. pylori* infection was more prevalent in those without IBS [22]. Contrary to these findings, *H. pylori* infection significantly increased the likelihood of health care-seeking behavior in those with IBS in a 10-yr longitudinal follow-up study [24]. Therefore, the aim of this study is to investigate whether *H. pylori* infection is associated with IBS.

## 2. Materials and Methods

### 2.1. Literature Search

We searched for articles published by December 2019 on PubMed, Cochrane Library, CINAHL and SCOPUS (which includes EMbase and ISI Web of Science) with keywords (*Helicobacter pylori* or *H. pylori* or *Helicobacter* infection or *pylori* infection) and (Irritable bowel syndrome or colon irritable). Articles published in English were only included for the search.

### 2.2. Inclusion Criteria and Exclusion Criteria

Inclusion criteria were as follows:

- Study population including patients with IBS regardless of diagnostic methods;
- Studies which evaluated *H. pylori* infection among study populations regardless of detection methods;
- Study design: cross-sectional studies, case-control studies and cohort studies.

Exclusion criteria are as follows:

- In vitro studies;
- Abstracts, review articles, letters, and case reports;
- Studies in which the subjects were children.

### 2.3. Data Extraction and Quality Evaluation

Articles were independently examined by 2 different reviewers. In the case of a discrepancy, second opinions were sought from the other reviewers, and reviewers discussed in order to reach the final decision. The extracted data were as follows: title, authors, published journal name, year of publication, country, study design, study objective, diagnostic methods for IBS, methods for *H. pylori* detection, the number of IBS patients, the number of controls, study period, mean age or median age and gender.

### 2.4. Quality Assessment

The Newcastle–Ottawa Scale (NOS) was used to assess the methodological quality of observational studies. The NOS includes 8 items judging 3 dimensions as follows: selection (0–4 stars), comparability (0–2 stars) and exposure for case-control studies or outcome for cohort studies (0–3 stars). The NOS ranges from 0 up to 9 stars. There are no definite criteria to determine a high-quality study in NOS. In this study, we presented the number of stars in each dimension of each study.

### 2.5. Statistical Analyses

We conducted a χ^2^ test of heterogeneity and calculated inconsistency index (I^2^) statistics. A value of I^2^ of 0%–25% represents insignificant heterogeneity, 26%–50% represents low heterogeneity, 51%–75% represents moderate heterogeneity and >75% represents high heterogeneity [25]. If significant heterogeneity existed among the articles, the random-effects model was selected. Otherwise, a fixed model was used for the analysis. The presence of publication bias was evaluated via Egger’s Test and Begg’s funnel plot [26]. Egger’s test is a regression method that uses the standardized estimate of the treatment effect as a dependent variable and its precision as an independent variable. In the Egger’s test, if *p* < 0.05, it means that there is a publication bias. For pooled estimate of binary data, odds ratio (OR) with its corresponding 95% confidence interval (CI) were calculated. A two-sided *p* value < 0.05 was considered statistically significant. All analyses were performed using Comprehensive Meta-analysis (version 3; Biostat, Inc., Englewood, NJ, USA).

## 3. Results

### 3.1. Characteristics of Eligible Studies

**Figure 1:**
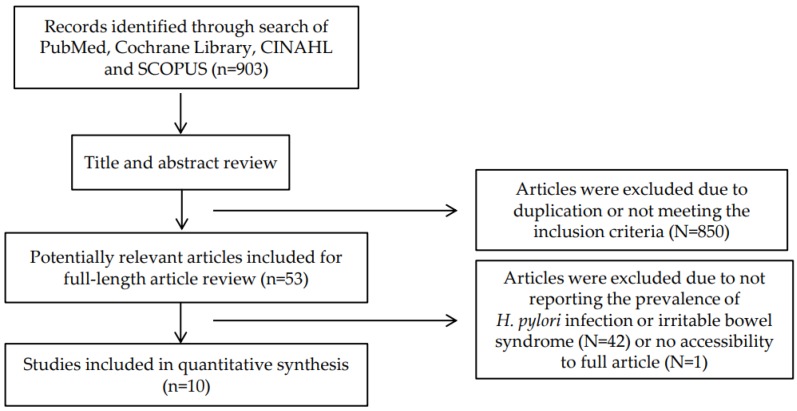
Flow chart of the search strategy.

Figure 1 showed the search strategy and selection flow of eligible articles. A total of 903 articles were identified through searches of Pubmed, Cochrane Library, CINAHL and SCOPUS. Among them, we excluded 850 articles including in vitro studies; and irrelevant types of articles, such as abstracts, letters and case-reports reviewing the titles and abstracts. After identifying 53 relevant articles, we reviewed the full length articles in detail except one article due to no accessibility to the full article. We excluded 42 articles since the number or prevalence of *H. pylori* infection and IBS were not demonstrated. Thus, a total of ten articles were selected for meta-analysis.

The characteristics of these eligible studies are summarized in Table 1. Four articles were cross-sectional studies, and the others were case-control studies. In regard to region, three studies in Europe, five in Asia, one in Africa and one in North America were conducted. The sample size ranged from 100 to 3148. The prevalence of IBS ranged from 5% to 52%. In regard to diagnostic criteria of IBS, Rome criteria were used in eight articles, and Manning criteria were used in one article. One study defined IBS based on the criteria suggested by authors [27]. Therefore, we conducted subgroup analysis only with studies conducted with Rome criteria among the diagnostic criteria of IBS.

### 3.2. Irritable Bowel Syndrome and H. pylori Infection

**Figure 2:**
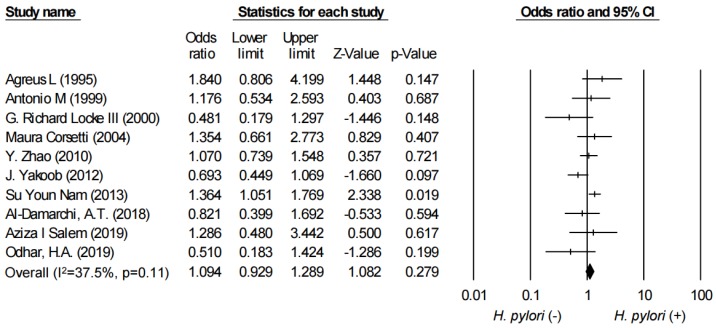
Forest plot evaluating the combined odds ratio (OR) between irritable bowel syndrome (IBS) and *Helicobacter pylori* infection.

In Figure 2, the combined OR between IBS and *H. pylori* infection was demonstrated in the forest plot. We applied the fixed-effect model for the analysis. The combined OR for *H. pylori* infection was 1.10 (95% CI: 0.93–1.29, I^2^ = 37.5%).

### 3.3. Subgroup Analysis

**Figure 3:**
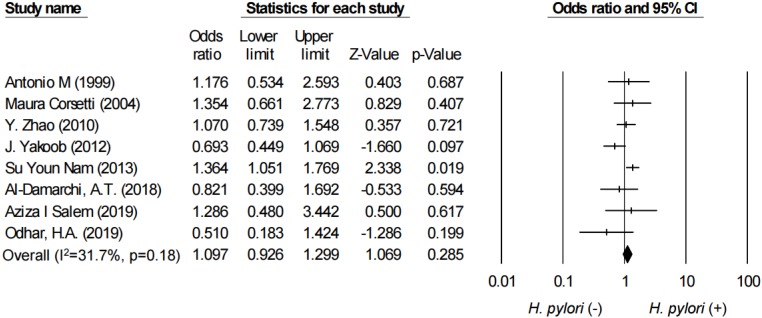
Forest plot evaluating the combined OR between IBS and according to Rome criteria and *H. pylori* infection.

We also performed subgroup analysis of articles which defined IBS according to Rome criteria. The combined OR was 1.10 (95% CI: 0.93–1.30, I^2^ = 31.7%), which is presented in Figure 3.

### 3.4. Evaluation for Publication Bias

**Figure 4:**
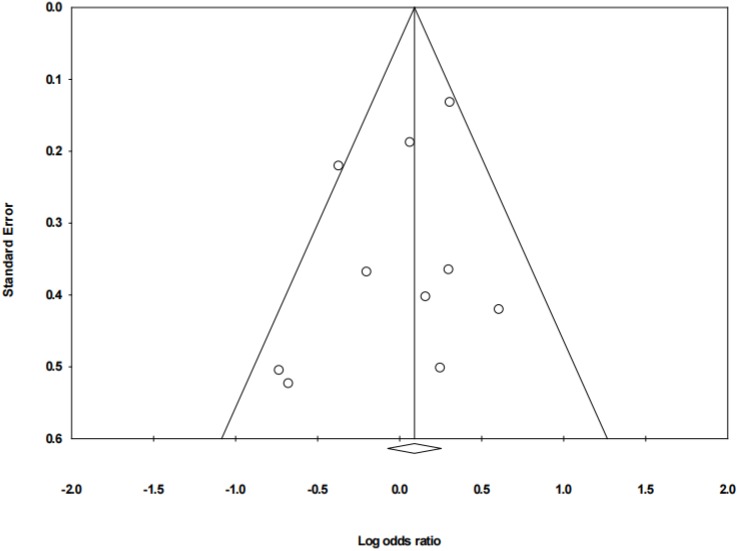
Funnel plot of included articles.

Egger’s test was performed to assess publication bias and showed no publication bias with *t* = −1.1789 (*p* = 0.272). The funnel plot was also drawn; see Figure 4.

## 4. Discussion

In this meta-analysis, we demonstrated that *H. pylori* infection was not significantly associated with IBS. The prevalence of *H. pylori* infection varied from 12.8% to 73.4% in the control group, and 9.7% to 72.1% in the IBS group.

Similar to our findings, individuals with IBS had increased likelihood of *H. pylori* infection in a recent meta-analysis by Ng QX et al., though there was no statistical significance (OR: 1.47, 95% CI: 0.90–2.40) [33]. Compared to that meta-analysis, there were some differences of search methods and inclusion criteria in this study. While we used Pubmed, Cochrane Library, CINAHL and SCOPUS which are representative databases for scientific research, Ng QX et al. included Google scholar and Chinese database, which might have lower quality articles. Besides, we only included English articles whose subjects were adults, though Ng QX et al. included Chinese articles and studies of children. In addition, we included more recent studies and performed subgroup analysis according to Rome criteria to lessen the hindering effect of different IBS diagnostic criteria. Last, heterogeneity among the articles was low in this study, whereas heterogeneity in the study by Ng QX et al. was high, as pointed out by those authors.

The associations with gastric or duodenal ulcer, gastric cancer and MALT lymphoma in *H. pylori* infection have already been well established [8,9,34]. Along with these clinical associations, the relationship between *H. pylori* and dyspepsia has been also studied [13,35]. In a case-control study with IBS patients, *H. pylori* infection increased the likelihood of dyspeptic symptoms, such as epigastric pain, postprandial abdominal fullness, early satiety, belching and nausea [36]. In a meta-analysis of 23 studies, the *H. pylori* infection was significantly associated with non-ulcer dyspepsia, and *H. pylori* eradication even improved dyspeptic symptoms by two times compared to no eradication [37]. According to the American College of Gastroenterology and the Canadian Association of Gastroenterology guidelines, noninvasive *H. pylori* testing is recommended for dyspepsia patients under the age of 60 and *H. pylori* eradication is also recommended if the result is positive [38]. Moreover, noninvasive *H. pylori* testing is initially recommended for those who complain of epigastric pain or postprandial fullness in a society where *H. pylori* is prevalent more than 10% [39].

IBS is one of the most common functional gastrointestinal disorders (FGIDs) along with functional dyspepsia [40]. The prevalence of the overlap between IBS and functional dyspepsia has been reported between 15% and 42% according to the different diagnostic criteria in each FGIDs. Besides, the likelihood for coexistence of IBS in subjects with dyspepsia was eight times higher compared with subjects without dyspepsia [41]. This strong association suggests that these two FGIDs have something common in their development. Alterations in gastrointestinal sensation and motor function; impaired intestinal mucosal integrity; infections; and psychological distress related to the brain-gut pathway have been suggested as possible mechanisms and causes for the development of IBS and functional dyspepsia [5,39,40]. In regard to infections, IBS-like symptoms have been reported to persist after acute bacterial or viral gastroenteritis in 10% to 30% of patients [42]. As one of possible infectious causes, *H. pylori* infection had been reported to be associated with gastric mucosa remodeling and an increase the neural responsiveness of smooth muscle in animal models [16].

According to the Rome Ⅳ criteria, IBS is diagnosed when recurrent abdominal pain related to defecation or altered stool form or stool frequency persists at least once per week in the previous three months with a duration of at least six months. Before diagnosing IBS, warning signs and possible disorders that can mimic IBS should be ruled out [5]. Before the introduction of Rome criteria, which form an expert consensus for diagnosing functional gastrointestinal disorders—first released in 1990 and updated periodically [43]—Manning criteria were used for the diagnosis of IBS [44]. According to the Manning criteria, IBS is diagnosed when any four of six symptoms related with abdominal pain or bowel movement are present [44]. Since the diagnostic criteria of IBS were diverse and different between the studies, we included all the related articles in the analysis regardless of how IBS was defined. Though we did not include the studies of chronic abdominal pain and altered bowel movement which resemble IBS but do not meet the IBS criteria, the number of studies included for this meta-analysis is small. This might underestimate the association between IBS and *H. pylori* infection. In a subgroup analysis of IBS according to same diagnostic criteria, the combined OR for *H. pylori* infection was relatively higher compared to that of all articles we included. However, statistical significance did not accompany the results.

There are some limitations that should be addressed. First, heterogeneity existed among the articles, though it was low. This might have originated from different study designs and methods, including how IBS was defined and how *H. pylori* was detected in each study. However, by performing subgroup analysis according to diagnostic criteria of IBS, we could lessen the effect of heterogeneity among articles. Second, we did not include the articles of IBS-like symptoms, as mentioned earlier. This can weaken the degree of association between *H. pylori* infection and IBS. Besides, we could not perform a full-length review of one article because of no accessibility to that journal, even though we tried to search through an overseas search program. Last, we could not identify the causal relationship between *H. pylori* infection and IBS due to the nature of meta-analysis of observational studies.

## 5. Conclusions

In conclusion, *H. pylori* infection was not significantly related with IBS based on this meta-analysis. Considering that current medical treatment of IBS is not enough to satisfy the patients and this can be linked to decreased overall quality of life, continuous research on effective IBS management should be encouraged and well-designed prospective studies are also needed to identify the relationship between *H. pylori* infection and IBS and to see whether *H. pylori* eradication helps improve IBS symptoms.

## Figures and Tables

**Table 1:** Characteristics of the studies included in meta-analysis.

Author	Year	Country	Design	*H. pylori* Diagnostic Method	IBS Diagnostic Criteria	Sample Size (Total No. of Study Participants)	No. of IBS	No. of Control	No. of *H. pylori* (+) in IBS Group	No. of *H. pylori* (+) in Control Group	Male (%)	Mean Age ± SD or Median Age (Range)	Quality Assessment (Newcastle-Ottawa Scale)
Agreus L [27]	1995	Sweden	Case-control	ELISA (Antibody-IgG for *H. pylori*)	Diagnostic criteria developed by authors	150	50	50	16	23	36	48 (22–80)	S2, C0, E2
Antonio M [28]	1999	Spain	Case-control	Antibody-IgG for *H. pylori*	Rome criteria	100	50	50	27	29	50	34.1 ± 7.4 in IBS, 35.6 ± 5.6 in control	S2, C1, E2
G. Richard Locke III [19]	2000	USA	Cross-sectional	ELISA (Antibody-IgG for *H. pylori*)	Manning et al.	148	35	77	9	11	42	31 (20–50)	S3, C2, O3
Maura Corsetti [29]	2004	Belgium	Cross-sectional	Endoscopic biopsy and stain	Rome Ⅱ	309	144	165	14	21	33	42.0 ± 0.8	S2, C0, O3
Y. Zhao [21]	2010	China	Cross-sectional	ELISA (Antibody-IgG for *H. pylori*)	Rome Ⅱ	3148	147	3001	106	2204	47.8	42.5 ± 15.2	S4, C2, O3
J. Yakoob [16]	2012	Pakistan	Case-control	Endoscopic biopsy	Rome Ⅲ	330	170	160	91	71	69 in IBS, 66 in control	40 ± 15 in IBS, 42 ± 14 in control	S2, C0, O3
Su Youn Nam [22]	2013	Korea	Cross-sectional	Endoscopic biopsy, UBT	Rome Ⅲ	2769	258	2511	106	1224	65.5 in IBS, 60.3 in control	45.3 ± 8.6 in IBS, 50.2 ± 9.9 in control	S3, C2, O3
Al-Damarchi, A.T [30]	2018	Iraq	Case-control	*H. pylori* stool antigen test	Rome Ⅳ	135	60	75	21	23	22 in IBS, 42 in control	33.5 ± 2.5 in IBS, 34.8 ± 3.1 in control	S2, C1, E1
Aziza I Salem [31]	2019	Egypt	Case-control	*H. pylori* Ag ELISA	Rome III	120	40	40	10	12	NA	28 ± 9.5 in IBS, 33.5 ± 13.9 in control	S2, C2, E2
Odhar, H.A. [32]	2019	Iraq	Case-control	Antibody-IgG for *H. pylori*	Rome Ⅳ	60	30	30	17	12	20 in IBS, 53.3 in control	23 (19–50) in IBS, 23 (19–55) in control	S2, C1, E1

Abbreviations: SD, standard deviation; S, selection; C, comparability; E, exposure; O, outcome; NA, not available.
